# Dihydrotestosterone Ameliorates Degeneration in Muscle, Axons and Motoneurons and Improves Motor Function in Amyotrophic Lateral Sclerosis Model Mice

**DOI:** 10.1371/journal.pone.0037258

**Published:** 2012-05-14

**Authors:** Young-Eun Yoo, Chien-Ping Ko

**Affiliations:** Section of Neurobiology, Department of Biological Sciences, University of Southern California, Los Angeles, California, United States of America; Medical College of Georgia, United States of America

## Abstract

Amyotrophic lateral sclerosis (ALS) is a lethal disease characterized by a progressive loss of motoneurons. The clinical symptoms include skeletal muscle weakness and atrophy, which impairs motor performance and eventually leads to respiratory failure. We tested whether dihydrotestosterone (DHT), which has both anabolic effects on muscle and neuroprotective effects on axons and motoneurons, can ameliorate clinical symptoms in ALS. A silastic tube containing DHT crystals was implanted subcutaneously in SOD1-G93A mice at early symptomatic age when decreases in body weight and grip-strength were observed as compared to wild-type mice. DHT-treated SOD1-G93A mice demonstrated ameliorated muscle atrophy and increased body weight, which was associated with stronger grip-strength. DHT treatment increased the expression of insulin-like growth factor-1 in muscle, which can exert myotrophic as well as neurotrophic effects through retrograde transport. DHT treatment attenuated neuromuscular junction denervation, and axonal and motoneuron loss. DHT-treated SOD1-G93A mice demonstrated improvement in motor behavior as assessed by rota-rod and gait analyses, and an increased lifespan. Application of DHT is a relatively simple and non-invasive procedure, which may be translated into therapy to improve the quality of life for ALS patients.

## Introduction

Amyotrophic lateral sclerosis (ALS) is a late onset neurodegenerative disease characterized by a progressive loss of motoneurons in the brain and spinal cord. The clinical symptoms of ALS include skeletal muscle weakness, atrophy and paralysis, which eventually lead to fatal respiratory failure within 2–5 years from the disease onset [1]. The majority of ALS cases are sporadic ALS caused by unknown etiology, while about 10% of ALS cases are the inherited form of ALS, called familial ALS (fALS). Recently, mutations in TDP-43 and FUS/TLS, which are DNA/RNA-binding proteins, were found in both types of ALS [2]. With a higher frequency, mutations in the human superoxide dismutase 1 (SOD1) gene are found in about 20% of fALS patients, and inserting these mutated human SOD1 genes into rodents have generated ALS animal models [3]. SOD1 mutations induce a gain of toxic function instead of a loss of enzymatic function, which converts reactive superoxide into hydrogen peroxide and water to reduce oxidative stress [4].

Furthermore, accumulating studies suggested that mutant SOD1-mediated toxicity in non-neuronal cells such as skeletal muscle and glial cells contribute to motoneuron degeneration in ALS [1], [5], [6], [7]. In accordance with the current notion that ALS is a non-cell autonomous disease and involves multiple cell types, inhibiting motoneuron death through Bax knock-out is not sufficient to prevent clinical symptoms of ALS, particularly in muscle [8]. Considering that muscle-related symptoms are closely related with the quality of the patient's life, targeting ALS-affected muscle may provide practical benefits to patients.

As a therapeutic intervention to reduce muscle weakness and atrophy, administration of androgens could be a potential strategy due to their roles in increasing muscle size and strength [9], [10], [11]. Androgens have been used for anabolic therapies to treat muscle wasting caused by chronic illness and aging [9]. However, it is not known whether androgen treatment can also increase muscle mass and strength in ALS patients who suffer from progressive muscle atrophy and associated motor defects. It is interesting to note that ALS patients show a lower level of free testosterone, which is a bioavailable form of androgen, compared with a non-ALS control group [12]. Since a low level of testosterone is associated with reduced muscle mass and strength [13], [14], it is possible that reduced androgen observed in ALS patients may contribute to muscle atrophy and weakness. Taken together, reduced androgen level may play a role in reducing the size and strength of skeletal muscle in ALS, and therefore androgen treatment might be one of the therapeutic approaches to alleviate muscle symptoms.

In addition to anabolic effects on muscle, androgen may directly benefit motoneurons through the highly expressed androgen receptors (ARs) in the ventral horn of the spinal cord [15]. The neuroprotective effects of androgens in promoting neuronal survival and neurite outgrowth have been found in the spinal motoneurons, and extensively in sexually dimorphic motoneurons such as the spinal nucleus of the bulbocavernosus [16]. Particularly, androgens enhance regeneration of the sciatic nerve after nerve crush by increasing the rate of nerve growth towards its target hindlimb muscles [17], [18]. Given that progressive motoneuron death and “dying-back” axonal retraction are the manifested pathology in ALS [19], [20], androgens may delay disease progression through its neuroprotective effects.

To examine a potential therapeutic effect of androgens in ALS, we administrated 5α-dihydrotestosterone (DHT), a metabolite of testosterone, to SOD1-G93A mice. The SOD1- G93A transgenic mouse is one of the extensively used animal models of ALS, which replicates pathological courses of ALS patients. SOD1-G93A transgenic mice demonstrate muscle atrophy and neuromuscular junction denervation from ∼50 days of age, ventral axon loss from ∼80 days of age, and motoneuron death from ∼100 days of age in a “dying-back” fashion [19], [20], and typically die at ∼140 days of age [21]. A silicon tubing containing DHT crystals was implanted subcutaneously in SOD1-G93A mice at early symptomatic age to mimic the time point when patients may receive treatments. In the present study, we found that DHT treatment ameliorates morphological defects in skeletal muscle, nerves, and motoneurons in SOD1-G93A mice. The morphological improvement was accompanied by enhanced motor performance and slightly increased survival duration.

## Materials and Methods

### Ethics Statement

All procedures related to laboratory animals were performed according to the US National Institutes of Health laboratory animal care guidelines. The protocol was approved by the Institutional Animal Care and Use Committee of the University of Southern California (protocol #: 10981). All efforts were made to minimize the suffering of animals.

### Animals

We used SOD1-G93A mutant mice with the congenic C57BL/6J background [B6.Cg-Tg (SOD1-G93A) 1Gur/J, Stock# 004435, The Jackson Laboratory], which exhibits a slightly longer lifespan with a more consistent phenotype and the smallest variation in survival than the extensively used hybrid line in C57Bl6/SJL background [21]. Because there are differences in disease progression, muscle size and strength in SOD1 mice caused by gender [21], [22], we administrated DHT only to male mice to avoid any ambiguity caused by gender, which will also avoid undesirable hormonal effects caused by androgen treatment in female mice. SOD1-G93A mutant mice were cross-bred with the same background mice expressing YFP in all motoneurons (thy1-YFP mice, B6.Cg-TgN (Thy1-YFPH) 2Jrs, Jackson Labs) to generate SOD1-G93A/YFP double transgenic mice for visualizing motor nerves [23]. Since this double transgenic does not elicit any changes in disease onset and progression in SOD1 mice [20], we used both SOD1-G93A and SOD1-G93A/YFP mice for behavioral studies. However, YFP expression in neurons may induce molecular changes related to cell stress responses and might modify abnormalities at neuromuscular junction (NMJ)s [24]. Therefore, it is important to compare molecular changes and NMJ innervation exclusively among either YFP-expressing animals or non-YFP animals. We used only SOD1-G93A/YFP mice for morphological and molecular assays, so the differences observed by DHT or orchidectomy were not affected by whether a mouse expressed YFP or not. Mice were housed in a 12-hour light-dark cycle, and given free access to food and water. Food and water were provided on the floor of the cage once the mice could not reach the food by themselves. For the morphological and molecular analyses, animals were sacrificed at postnatal day 120 (P120), which was the symptomatic age showing marked reduction in muscle weight and motor performance. The mice used for the behavioral and survival analyses were sacrificed at the end-stage when a mouse was not able to right itself within 30 s when placed on one side. Mice were anesthetized via an intraperitoneal (i.p.) injection with a mixture of xylazine (10 mg/kg body weight) and Ketamine (80 mg/kg body weight).

### DHT implant

Crystalline 5α-dihydrotestosterone (DHT) (Sigma) was administered in the form of silastic implant. One-cm length of silastic medical tubing (0.078 cm-inner diameter/0.0125 cm- outer diameter; Dow Corning Corporation) was filled with 5 mg of DHT, and both ends were sealed with a silastic medical-grade adhesive (Dow Corning). Silastic tubing without DHT was used as a control. Before implanting the silastic tube, the tubes were equilibrated in saline overnight. DHT-filled or empty silastic tubes were implanted subcutaneously in the backs of anesthetized male SOD1-G93A mice at P75. Based on the method of calculating release dosage based on the surface area of the DHT-filled silastic tube, the estimation of its plasma concentration was ∼500 ng/dl plasma, which is higher than normal physiological level [25].

### Orchidectomy surgery

Mice were anesthetized via an intraperitoneal (i.p.) injection with a mixture of Rompun (8 mg/kg body weight) and Ketamine (80 mg/kg body weight). The scrotum was shaved, and a small incision (0.5–1 cm) at the ventral midline was made. The tunica was exposed by pushing skin away, and was pierced. The spermatic artery was sutured, before removing testis to avoid bleeding. After removing both testes, one or two stitches were made to close the incision, and animals recovered on a 37°C heating pad. Buprenex was administered (0.1 ml/20 gram, i. p.) for analgesia after surgery. Surgical instruments were sterilized by an autoclave prior to surgeries.

### Histological examination of muscle and neuromuscular junctions

The tibialis anterior (TA) and diaphragm (DIA) muscles were dissected from SOD1-G93A/YFP double transgenic mice that express YFP in motor nerves, and fixed with 4% paraformaldehyde for 30 min. To measure cross sectional area, total number of muscle fibers, and the size of single muscle fiber, TA was rinsed in phosphate-buffered saline (PBS) three times for 10 min and cryo-protected in 30% sucrose overnight. TA muscles were embedded in Tissue Tek O.C.T (Sakura Finetek), flash-frozen in supercooled isopentane, and sectioned (16 µm thickness) on a cryostat. The mid-belly areas were sectioned, and stained with monoclonal anti-myosin (Sigma). The cross sectional areas of muscle fibers were determined by using Image J (NIH Image). To examine NMJ innervation, TA and DIA muscle were rinsed in PBS, and whole muscles were teased and stained with Alexa-594-alpha-bungarotoxin (BTX) (Invitrogen, 1∶2000) for acetylcholine receptor (AChR) clusters. For a blinded analysis, each treatment was coded by the investigator who collected muscles, and another investigator examined 400–500 NMJs per muscle for NMJ quantification without knowing to which group a particular animal belonged.

### Histological examination of motor axons

Ventral roots were collected from the lumbar segment 4 (L4) that was fixed with 4% paraformaldehyde in 0.1 M phosphate-buffered saline. Phrenic nerves attached to the diaphragm muscle were collected by cutting the entry part of the nerves to the muscle and fixed with 4% paraformaldehyde. Both ventral roots and phrenic nerves were washed in 0.1 M phosphate buffer, and further fixed with 1% osmium tetroxide for 2 h, dehydrated, and embedded in Epon plastic (EM Sciences, Cincinnati, OH). Cross-sections (1 µm-thickness) were stained with toluidine blue and observed under light microscopy to measure axon numbers and calibers with Image J (NIH image).

### Histological examination of α-motoneurons

Animals were perfused with saline followed by 4% paraformaldehyde in 0.1 M PBS. After the lumbar segments (L3-L5) were dissected, they were rinsed in PBS, and cryo-protected in 30% sucrose overnight. Lumber segments were embedded in Tissue Tek O.C.T (Sakura Finetek), flash-frozen in supercooled isopentane, and sectioned (25 µm-thickness) on a cryostat. Every 5^th^ section from the serial cross-sections was stained with anti- choline acetyltransferase (ChAT) antibody (Chemicon, 1∶200) to label α-motoneurons (determined by cell body size ≥250 µm^2^). About 15–20 sections summing to about 200–300 motoneurons for each spinal cord were counted.

### Quantitative RT-PCR analysis

Total RNA was extracted from the tibialis anterior muscle by using TRIzol reagent (Invitrogen), and treated with DNase by using DNA-free kit (Ambion). Two µg of total RNA measured with a spectrophotometer at 260 nm absorbance was reverse-transcribed using the SuperScript III (Invitrogen) for a synthesis of complementary DNA (cDNA). cDNA was diluted in 1∶20, and 5 µl of diluted cDNA was used in each 20 µl PCR reaction. Real-time quantitative PCR (qPCR) was performed with SYBR green dye using DNA Engine Opticon 2 system (Bio-Rad) with a program of 40 cycles of 95°C 20 sec, 60°C 20 sec, 72°C 30 sec. The relative expression level for each gene was calculated using the 2 -ΔΔCt method [26], and all expression values were normalized to housekeeping genes, such as glyceraldehyde-3-phosphate dehydrogenase (GAPDH) or β-actin.

The sequence of the primers as follows:

Insulin-like growth factor (IGF)-1: forward, 5′- CTGGACCAGAGACCCTTTGC, reverse, 5′- GGACGGGGACTTCTGAGTCTT; IGF-2: forward, 5′- GTGCTGCATCGCTGCTTAC, reverse, 5′- ACGTCCCTCTCGGACTTGG; Muscle ring finger (MuRF)-1: forward, 5′-ACCTGCTGGTGGAAAACATC, reverse, 5′-CTTCGTGTTCCTTGCACATC; GAPDH: forward, 5′- TGCATCCTGCACCACCAACT, reverse, 5′- ATGCCTGCTTCACCACCTTC; β-actin: forward, 5′- GGCTGTATTCCCCTCCATCG, reverse, 5′- CCAGTTGGTAACAATGCCATGT.

### Behavioral tests

Mice were monitored every 5 days for measuring their body weights, and perform behavioral tests. For the grip-strength analysis, mice were allowed to grasp a triangular pull bar from the grip strength meter (Columbus Instruments). Once a mouse made a stable grip with the pull bar, the mouse was slowly pulled away from the pull bar until the grip was released. Maximum tension of the pull bar was digitally recoded with the grip strength meter. Five measurements were taken from each animal, and the mean values were used for statistical analysis. For the rota-rod test, a mouse was placed on a rotating rod that rotates at a speed of 11 rpm, and the latency until a mouse falls from the rod was recorded. The longest latency (in sec) in 3 trials was recorded. For the footprint analysis, hindlimbs were placed into non-toxic paints and the animal was allowed to walk on a 50 cm length of paper, and their continuous locomotion was recorded. Step length was measured by dividing the walking distance by the number of steps taken within the walking distance.

### Statistical Analysis

All data are expressed as the mean ± SEM. All statistical analyses were performed on PRISM 5.01 software (Graph-Pad, San Diego), using two-tailed student's t-tests, except a two-way ANOVA for analyzing muscle fiber distribution and the rota-rod test. Kaplan-Meier method was used to create survival curves, and log-rank (Mantel-Cox) test was used for comparing the difference in survival. Significance was defined as *p*<0.05.

## Results

### DHT implant successively increases androgen level in wild-type and SOD1-G93A mice

**Figure 1:**
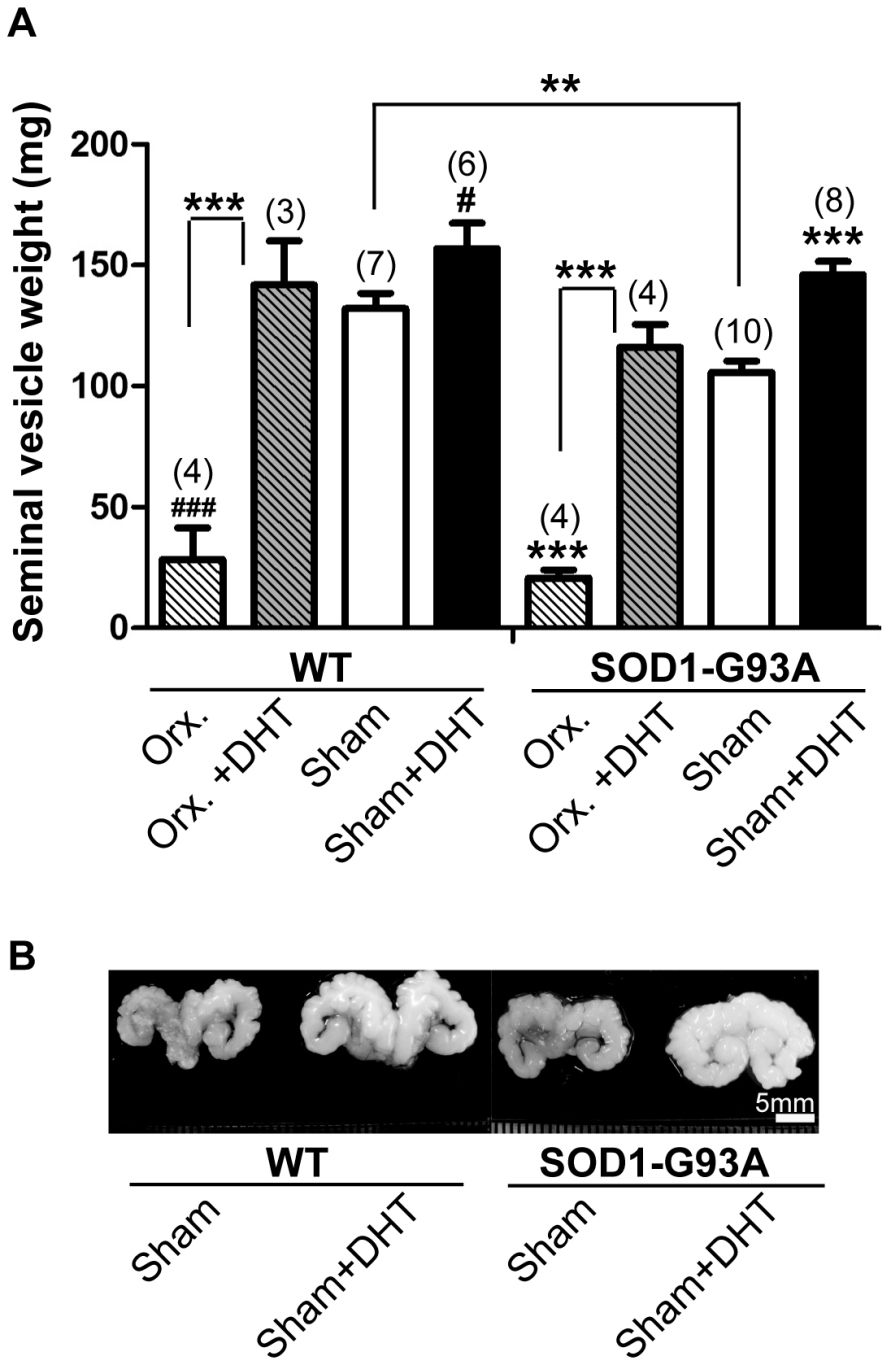
DHT increases whereas orchidectomy decreases the seminal vesicle weight in wild-type and SOD1-G93A mice. **A:** The weight of seminal vesicles was measured after removing the adhering tissue and fluid. Wild type (WT) mice and SOD1-G93A (SOD1) mice were implanted with either a DHT-filled or an empty silastic tube. Orchidectomy and/or silastic tube implant was performed at postnatal day 75 (P75), and the seminal vesicle weight was measured at P120. DHT-treated WT mice showed a 19% increase in seminal vesicle weight (156±10.6 mg, *p* = 0.042) compared with control WT mice (132.1±6.4 mg). In SOD1 mice, the DHT-filled silastic tube also increased the seminal vesicle weight by 38% (146.1±5.5 mg, *p* = 0.0003) compared with control SOD1 mice (105.8±4.6 mg). Conversely, orchidectomy decreased the seminal vesicle weight in both WT mice (28.4±13 mg, *p*<0.0001) and SOD1 mice (20.8±3.5 mg, *p*<0.0001) compared with control WT and SOD1 mice, respectively. SOD1 control mice showed 20% reduced seminal vesicle weight compared with WT control mice (*p* = 0.0039). Sample size is indicated in ( ) for each group. Data are mean ± SEM. # *p*<0.05, ### *p*<0.001 (compared with age-matched WT mice), ** *p*<0.01, ***p<0.001 (compared with control SOD1 mice). **B:** WT and SOD1 mice were implanted with either a DHT-filled or an empty silastic tube at P75, and the seminal vesicles were obtained at P120. Representative pictures of the seminal vesicles are shown. Scale bar = 5 mm.

We used a DHT-filled silastic tube, which is an extensively used method to release hormones systemically [17], [25], to increase the concentration of DHT in our study. We thus first tested whether the implant can successively administrate DHT by implanting DHT-filled tubes in wild-type mice at postnatal day 75 (P75). Empty silastic tubes were also implanted in age-matched littermates, which served as a control to exclude any possible artifact caused by the implant surgery. To examine whether the DHT implant increased androgen concentration, the weight of seminal vesicles was measured at P120. Compared to a direct measurement of DHT concentration in serum, which can be affected by anesthesia and stress during blood collection [27], [28], measuring the seminal vesicle weight is a sensitive, albeit indirect, way to assess the androgen level [13]. As shown in Fig. 1A and B, DHT treatment in wild-type mice caused about a 20% increase in the seminal vesicle weight compared with control wild-type mice. As a reverse way to examine the sensitivity of the seminal vesicle to androgen concentration, we attempted to decrease androgen concentration by performing orchidectomy in wild-type mice, and checked whether the seminal vesicle weight decreased. After removing at P75 both testicles, which generate androgens, we found a 79% reduction in the seminal vesicle weights in wild-type mice (Fig. 1A), indicating that the seminal vesicle weight is sensitive to androgen concentration. DHT implant in orchidectomized wild-type mice recovered the seminal vesicle weight to the normal level (Fig. 1A), which confirms a successful administration of DHT through the implant.

Since it was reported that ALS patients show a lower level of the bioavailable form of testosterone compared with a non-ALS control group [12], we examined whether the seminal vesicle weight of SOD1-G93A mice is smaller than that of wild-type mice. As shown in Fig. 1A, SOD1-G93A mice showed a 20% lower seminal vesicle weight compared with wild-type mice (SOD1-G93A: 105.8±4.6 mg, WT: 132.1±6.4 mg, *p* = 0.0039), which reflects a lower androgen level in SOD1-G93A mice compared with wild-type mice. To test whether DHT implant can also increase androgen level in SOD1-G93A mice, we implanted DHT-filled silastic tube at P75, and found that the DHT implant increased the seminal vesicle weight in SOD1-G93A mice by 38% (SOD1-G93A+DHT: 146.1±5.5 mg, *p* = 0.0003, Fig. 1A and B). Conversely, orchidectomy in SOD1-G93A mice caused an 80% reduction in seminal vesicle weight (SOD1-G93A+Orx.: 20.8±3.5 mg, *p*<0.0001) compared with control SOD1-G93A mice, and DHT treatment to orchidectomized SOD1-G93A mice was able to restore the seminal vesicle weights comparable to control SOD1-G93A mice (SOD1-G93A+Orx.+DHT: 116.1±9.5 mg, Fig. 1A). Taken together, SOD1-G93A mice demonstrate lower seminal vesicle weight, which reflects the lower concentration of androgens compared with wild-type mice. Also, we confirmed that the DHT implant could successively release DHT, which was reflected by the increase in seminal vesicle weight.

### DHT attenuates skeletal muscle atrophy in SOD1-G93A mice

**Figure 2:**
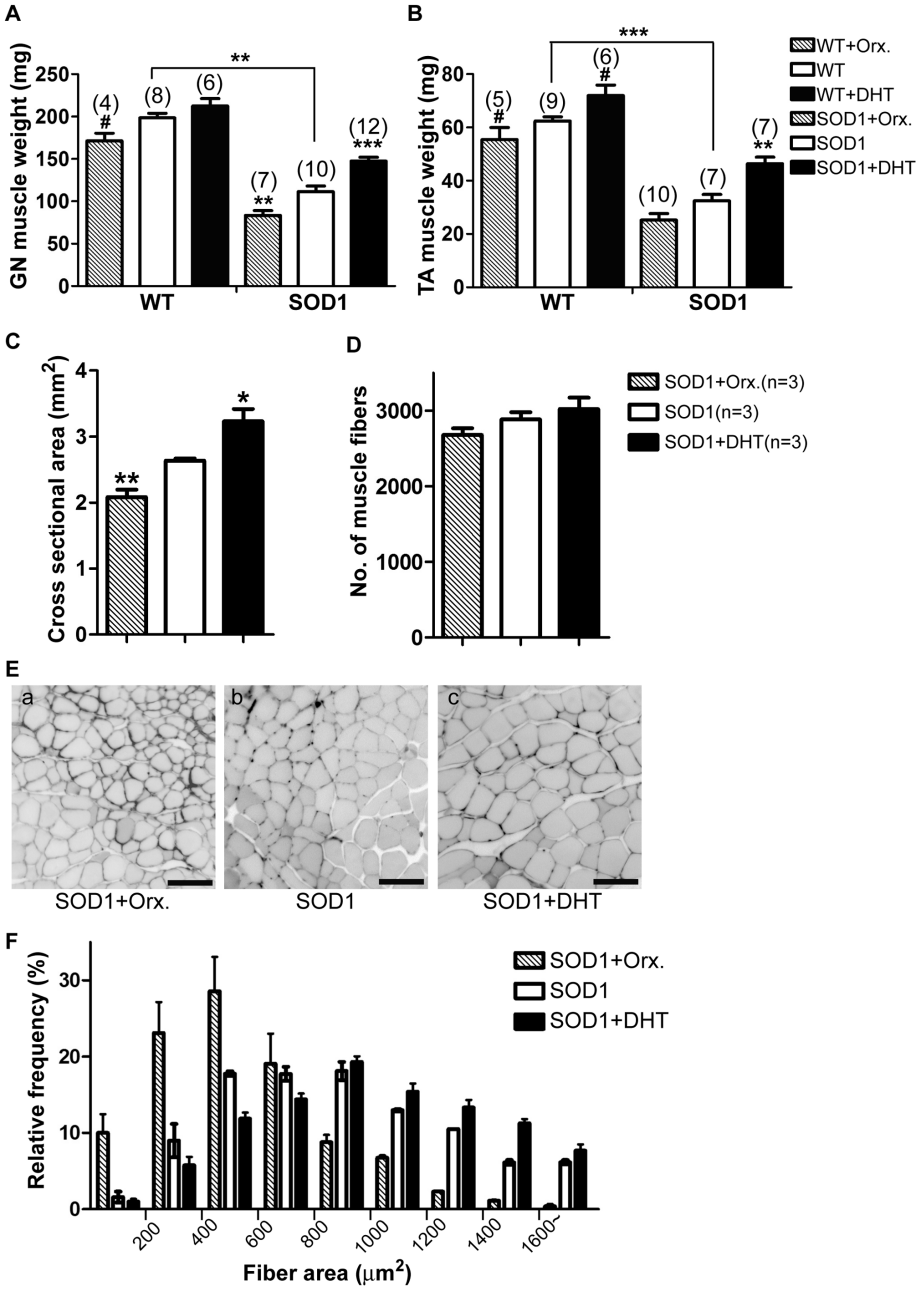
DHT increases whereas orchidectomy decreases the weight and cross sectional area of hindlimb muscles. WT and SOD1 mice were implanted with either a DHT-filled or an empty silastic tube, or orchidectomized at P75, and the morphological analyses were made at P120. **A:** In WT mice, DHT increased the GN weight by 7% (212.4±8.8 mg), whereas orchidectomy decreased it by 14% (171.4±9.0 mg) compared with control WT mice (198.4±5.5 mg). In SOD1 mice, DHT increased the GN weight by 32% (147.5±4.5 mg, *p* = 0.00017), whereas orchidectomy decreased it by 25% (83.4±5.7 mg, *p* = 0.0086) compared with control SOD1 mice (111.4±6.6 mg). **B:** In WT mice, DHT increased the weight of TA muscle by 12% (71.2±2.5 mg), whereas orchidectomy decreased it by 12% (56.1±3.6 mg) compared with control WT mice (63.6±1.5 mg). In SOD1 mice, DHT increased the weight of TA muscle by 43% (46.3±2.4 mg, *p* = 0.0017), whereas orchidectomy decreased it by 22% (25.2±2.4 mg, *p* = 0.05) compared with control SOD1 mice (32.5±2.5 mg). Sample size is indicated in ( ) for each group. ## *p*<0.01, ### *p*<0.01 (compared with age-matched WT mice), ** *p*<0.01, ***p<0.001 (compared with control SOD1 mice). **C:** DHT increased the cross-sectional area of TA muscle by 22.3% (3.23±0.19 mm^2^, n = 3, *p* = 0.034), whereas orchidectomy decreased it by 20.8% (2.09±0.11 mm^2^, n = 3, *p* = 0.008) compared with control SOD1 mice (2.64±0.03 mm^2^, n = 3). **D:** DHT did not cause a significant increase in the muscle fiber number (4.8% increase, 3020.7±152.2, *p* = 0.49). Likewise, orchidectomy did not cause a significant decrease in the muscle fiber number (7.0% decrease, 2681.0±86.9, *p* = 0.19) compared with control SOD1 mice (2883.7±97.0). **E:** Representative pictures of the cross sectional area of TA muscles are shown. Scale bar = 50 µm. **F:** Distribution of the area of single muscle fiber is shown. Per TA muscle, 600–900 muscle fibers were measured, and 3 TA muscles per each treatment group were used for the analysis of muscle fiber area. DHT treatment shifted the area of muscle fibers toward larger areas (1070.8±39.8 µm^2^, *p* = 0.032), whereas orchidectomy shifted it toward smaller areas (729.1±61.9 µm^2^, *p* = 0.023) compared with control SOD1 mice (904.7±26.6 µm^2^). Data are mean ± SEM. *p*<0.001 (2 way-ANOVA).

To examine whether DHT treatment attenuates skeletal muscle atrophy in SOD1-G93A mice, we measured the weight of gastrocnemius (GN) and tibialis anterior (TA), which are vulnerable muscles in ALS [29], [30]. Muscle atrophy in GN and TA muscles was observed in SOD1-G93A mice by showing a 44% and a 49% decreased muscle weight, respectively, compared with wild-type mice (Fig. 2A and B). DHT treatment increased the GN muscle weight by 32% (SOD1-G93A+DHT: 147.5±4.5 mg, SOD1-G93A: 111.4±6.6 mg, *p*<0.001, Fig. 2A), and the TA muscle weight by 43% (SOD1-G93A+DHT: 46.3±2.4 mg, SOD1-G93A: 32.5±2.5 mg, *p* = 0.0017, Fig. 2B). Conversely, orchidectomized SOD1-G93A mice with lower androgen concentration showed a decrease in the muscle weight of GN and TA by 25% and 22%, respectively (GN: 83.4±5.7 mg, TA: 25.2±2.4 mg, Fig. 2A and B). A similar pattern of increase or decrease in muscle weight by DHT treatment or orchidectomy, respectively, was observed in wild-type mice as well (Fig. 2A and B).

To examine whether the increase in muscle weight by DHT treatment is associated with the increase in cross-sectional area, we measured the cross-sectional area of TA muscle. Compared to control SOD1-G93A mice, DHT-treated SOD1-G93A mice demonstrated 22.3% increase in cross-sectional area in TA muscles (SOD1-G93A+DHT: 3.23±0.19 mm^2^, SOD1-G93A: 2.64±0.03 mm^2^, *p* = 0.034, Fig. 2C). Similar to the decrease in muscle weight by orchidectomy, we found that orchidectomy decreased the cross-sectional area of TA muscle (SOD1-G93A+Orx.: 2.09±0.11 mm^2^, *p*<0.01, Fig. 2C). To further examine whether the differences in cross-sectional area are caused by the differences in the number and/or the area of muscle fibers, we analyzed total muscle fiber numbers and muscle fiber areas of TA muscles. As shown in Fig. 2D, the total numbers of muscle fibers in TA muscles were not significantly different among orchidectomized, control, and DHT-treated SOD1-G93A mice, although there was a trend of containing more muscle fibers in association of higher androgen concentration. However, DHT treatment significantly increased the muscle fiber area, while orchidectomy decreased the muscle fiber area in TA muscles (Fig. 2E and F). There was a shift toward a larger area of single muscle fiber in DHT-treated SOD1-G93A mice, and a shift toward a smaller muscle fiber area in orchidectomized SOD1-G93A mice compared with control SOD1-G93A mice (*p*<0.01, Fig. 2F).

### DHT increases body weight and muscle strength in SOD1-G93A mice

**Figure 3:**
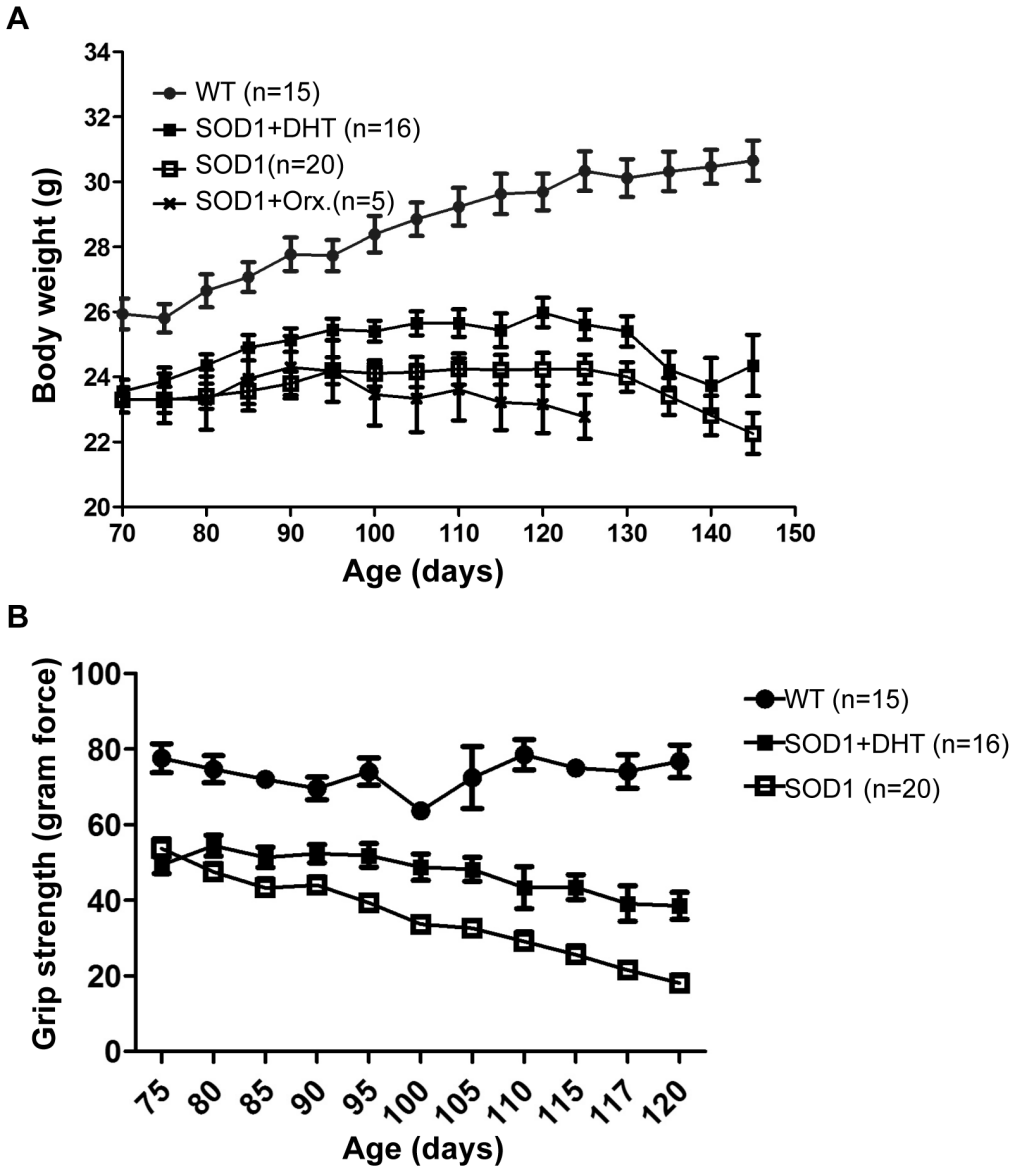
DHT increases body weight and muscle strength in SOD1 mice. **A:** DHT-treated SOD1 mice showed heavier body weight compared with control SOD1 mice (*p*<0.001), although it was still lower than the weight of WT mice throughout all time points. Orchidectomized SOD1 mice demonstrated reduced body weights compared with control SOD1 mice (*p* = 0.045). Data are mean ± SEM. **B:** The grip-strength meter was used to assess the muscle strength, and the maximum tension generated by the grip of a mouse on the pull bar was recorded. SOD1 mice exhibited diminished grip-strength compared with WT mice throughout all time points examined (*p*<0.0001). DHT-treated SOD1 mice showed stronger grip-strengths compared with control SOD1 mice (*p*<0.001), and the gap between these two groups gradually increased as age advanced. Data are mean ± SEM.

To examine whether increased muscle weight and size through a DHT implant was associated with the increase in body weight, we measured the body weight of SOD1-G93A mice. As shown in Fig. 3A, DHT-treated SOD1-G93A mice demonstrated heavier body weights compared with control SOD1-G93A mice (*p*<0.001), although it was not able to be rescued to the level of wild-type mice. Decreased androgen concentration in SOD1-G93A mice by orchidectomy led to further reduction in body weights compared with control SOD1-G93A mice (*p* = 0.045, Fig. 3A).

Increased muscle mass mediated by androgens has been shown to be correlated with increased muscle strength [11], [31], [32]. Therefore, we examined whether increased muscle mass through DHT treatment is accompanied by enhanced muscle strength in SOD1-G93A mice. To assess muscle strength, we employed the grip-strength meter, which measures the maximum tension generated by the grip of a mouse on the pull bar. As shown in Fig. 3B, SOD1-G93A mice exhibited 40% reduced grip-strength compared with wild-type mice at P90, and the grip-strength gradually weakened to reach only 20% of wild-type grip-strength at P120. DHT treatment increased grip-strength by about 20% stronger at P90 and 200% stronger at P120 in SOD1-G93A mice (Fig. 3B).

### DHT increases the expression of insulin-like growth factor (IGF) -1 and -2, whereas decreases the expression of MuRF-1 in skeletal muscle of SOD1-G93A mice

**Figure 4:**
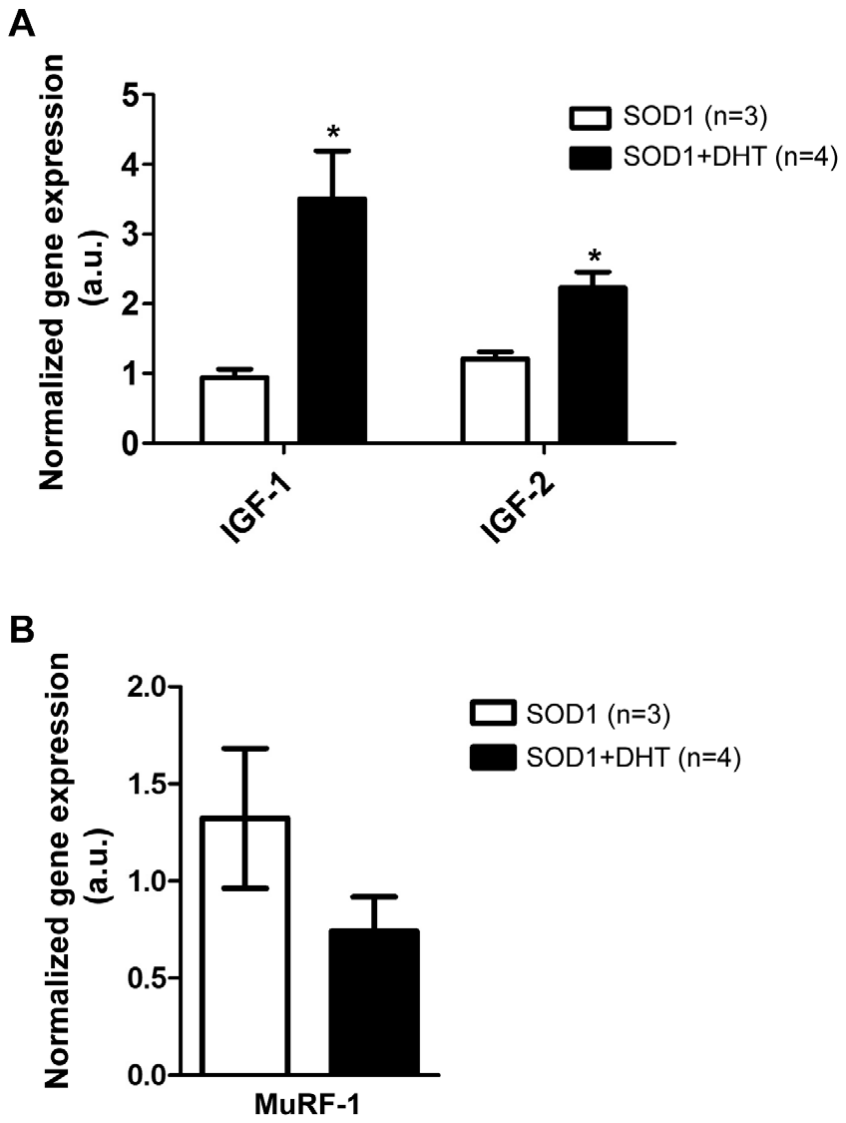
DHT increases the expression of insulin-like growth factor -1 and -2, while decreases MuRF-1 expression. **A:** The TA muscles were collected from DHT-treated SOD1 and control SOD1 mice at P120 to check the expression of insulin-like growth factor (IGF) -1 and IGF-2 through quantitative RT-PCR. DHT-treated SOD1 mice showed increased expression of IGF-1 and IGF-2, by approximately 4-fold (*p* = 0.0261), and 2-fold (*p* = 0.015), respectively, compared with control SOD1 mice. *p<0.05. **B:** By using quantitative RT-PCR, we found that DHT-treated SOD1 mice showed a trend of decreased expression of MuRF-1 by 44% compared with control SOD1 mice (*p* = 0.198).

Increased skeletal muscle mass through androgens is known to be mediated by insulin-like growth factor (IGF-1), which induces myoblast proliferation, differentiation, and hypertrophy [33], [34], [35], [36]. To examine whether DHT increases IGF-1 expression in skeletal muscle of SOD1-G93A mice, we performed quantitative RT-PCR. As shown in Fig. 4A, DHT treatment increased the expression of IGF-1 by about 4-fold (*p* = 0.0261) in the TA muscle compared with control SOD1-G93A mice at P120. Similarly, the expression level of IGF-2, which plays a similar role in muscle to IGF-1 [37], [38], was also increased by about 2-fold (*p* = 0.015) in DHT-treated SOD1-G93A mice compared with control SOD1-G93A mice (Fig. 4A).

Another mechanism associated with muscle size is the ubiquitin–proteasome system, which leads to degradation of muscle proteins. Especially, Muscle RING Finger1 (MuRF-1), a muscle-specific ubiquitin ligase, is required to induce rapid muscle atrophy [39], [40]. Therefore, we tested whether ameliorated muscle atrophy in DHT-treated SOD1-G93A mice was associated with the decreased expression of MuRF-1. By using quantitative RT-PCR, we found that there was a trend that DHT treatment decreased the expression of MuRF-1 by 43.9% compared with control SOD1-G93A mice (*p* = 0.198, Fig. 4B), which implies that molecular pathway that triggers muscle atrophy might be attenuated by DHT treatment. In summary, myotrophic pathway mediated by IGF-1 and -2 was enhanced, while muscle wasting pathway mediated by MuRF-1 is attenuated in DHT-treated SOD1-G93A mice compared with control SOD1-G93A mice.

### DHT improves the neuromuscular junction (NMJ) innervation in SOD1-G93A mice

**Figure 5:**
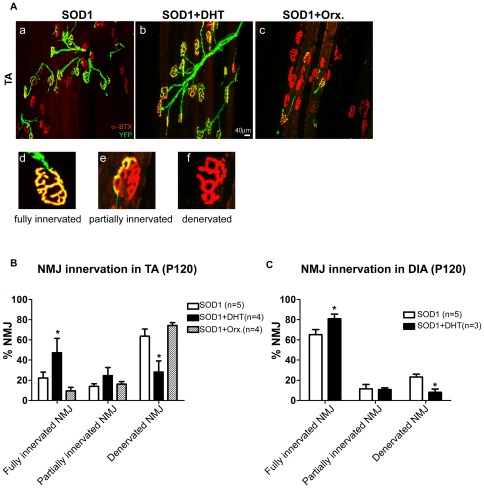
DHT ameliorates denervation at neuromuscular junctions SOD1 mice. SOD1/YFP double transgenic mice expressing yellow fluorescence protein (YFP) in all motor nerves were implanted with either a DHT-filled or an empty silastic tube, or orchidectomized at P75, and the TA muscle and the diaphragm (DIA) muscle were collected at P120, and stained with anti-α-bungarotoxin to label post-synaptic acetylcholine receptor (AChR) clusters. **A:** When a pre-synaptic nerve terminal (in green) fully overlaps with the post-synaptic AChR clusters (in red), the neuromuscular junction (NMJ) is defined as a “fully innervated NMJ” (d). However, if a nerve terminal is partially overlapped with AChR, or is completely absent, leaving only AChR, the NMJ is defined as a “partially innervated NMJ” (e) or a “denervated NMJ” (f), respectively. DHT-treated SOD1/YFP mice (b) showed improved NMJ innervation in the TA muscle compared with control SOD1/YFP mice (a). However, orchidectomy in SOD1/YFP mice aggravated denervation at NMJs (c). **B:** Quantification of NMJs at P120 in the TA muscle of DHT-treated, control, and orchidectomized SOD1/YFP mice is shown. Compared to control SOD1/YFP mice, which showed 22.3±5.7% of fully innervated NMJs, DHT-treated SOD1/YFP mice showed 47.3±14.1% of fully innervated NMJs. Orchidectomized SOD1/YFP mice showed only 9.6±3.4% of fully innervated NMJs. **C:** Quantification of NMJs at P120 in the DIA muscle of DHT-treated, and control SOD1/YFP mice is shown. Compared to SOD1/YFP mice, which showed 65.2±5.6% of fully innervated NMJs, DHT-treated SOD1/YFP mice showed 81.0±5.5% of fully innervated NMJs. Data are mean ± SEM. *p<0.05.

Together with muscular atrophy, denervation at the NMJs is a prominent symptom found in skeletal muscle of ALS [19], [29]. To visualize NMJs, we used double transgenic mice SOD1-G93A/YFP, which express fluorescent protein in all motor nerves. As shown in Fig. 5A and B, SOD1-G93A/YFP mice showed a 77% denervated or partially innervated NMJs in TA muscle at P120. DHT treatment decreased the denervated NMJs by 56% in the TA muscle, and increased the fully innervated NMJs by 2-fold (*p* = 0.02, *p* = 0.049, respectively, Fig. 5Ab and B). Conversely, decreasing androgen concentration through castration showed about 70% less fully innervated NMJs in the TA muscle in orchidectomized SOD1-G93A/YFP mice compared with control SOD1-G93A/YFP mice (*p* = 0.058, Fig. 5Ac, and B). We further examined denervation in the diaphragm muscle (DIA) that causes fatal respiratory failures in ALS patients. As shown in Fig. 5C, DHT-treated SOD1-G93A/YFP mice exhibited a 24% increase in fully innervated NMJs, and a 64% decrease in denervated NMJs compared with control SOD1-G93A/YFP mice (*p* = 0.0039, *p* = 0.014, respectively). Taken together, DHT treatment ameliorates NMJ denervation in SOD1-G93A mice, which may contribute to improved muscle phenotype and motor function.

### DHT ameliorates axonal degeneration in SOD1-G93A mice

**Figure 6:**
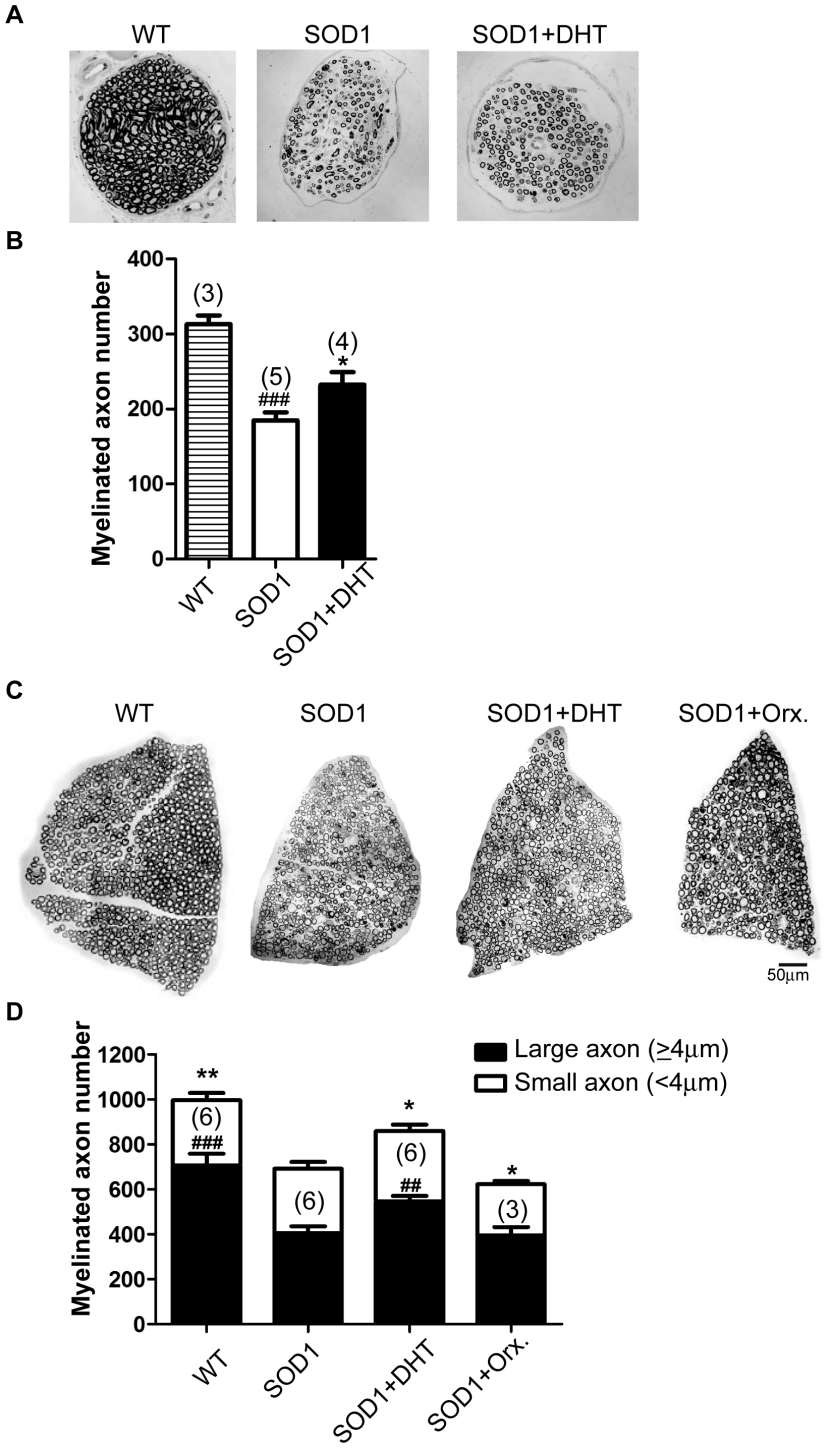
DHT attenuates axonal loss in the phrenic nerve and ventral root of the spinal cord. **A:** The phrenic nerve at the entry of the diaphragm muscle was sectioned to observe myelinated axons at P120. Representative pictures of the phrenic nerve from WT, control SOD1, and DHT-treated SOD1 mice are shown. **B:** Quantification of myelinated axon number in the phrenic nerves is shown. The number of myelinated axons in the phrenic nerve of SOD1 mice (184.6±10.9) is about 40% less compared with WT mice (313.0±11.5, *p* = 0.0003). DHT-treated SOD1 mice showed 26% more myelinated axons (232.3±16.8, *p* = 0.043) compared with control SOD1 mice. Sample size is indicated in ( ) for each group. *p<0.05 (compared with control SOD1 mice), ### *p*<0.001 (compared with WT mice). **C:** Representative cross-sectional pictures of the ventral roots of the spinal cord lumbar 4 segment from WT, control SOD1, DHT-treated SOD1, and orchidectomized SOD1 mice at P120 are shown. **D:** Quantification of myelinated axon number in the ventral root of the lumbar 4 spinal cord is shown. The total number of myelinated axons in control SOD1 mice (691.2±43.6) is about 30% less compared with that in WT mice (996.5±58.5, *p* = 0.0012). DHT-treated SOD1 showed 24% more total myelinated axon number compared with control SOD1 mice (859.6±53.4, *p* = 0.013). Especially, the number of the large caliber axons (≥4 µm) were 43% less in SOD1 mice (404.2±31.1) compared with WT mice (706.5±51.6, *p* = 0.0005). DHT-treated SOD1 mice showed 36% more large caliber axons (≥4 µm) (547.8±22.2, *p* = 0.006) compared with control SOD1 mice. Orchidectomized SOD1 mice showed 10% less total myelinated axon number compared with control SOD1 mice (622.3±29.6, *p* = 0.01). Sample size is indicated in ( ) for each group. Data are mean ± SEM. *p<0.05, ** *p*<0.01 (compared with the total myelinated axon numbers in SOD1 control), ##*p*<0.01, ### *p*<0.001 (compared with the number of large caliber axons in SOD1 control).

To further examine whether the beneficial effect of DHT at NMJs is accompanied by improved morphology in nerves, we checked the phrenic nerve at the entry of the diaphragm muscle. In SOD1-G93A mice, we found about a 40% loss in myelinated axons in the phrenic nerves compared with wild-type mice at P120 [SOD1-G93A:184.6±10.9, WT: 313.0±11.5, *p* = 0.0003 (Fig. 6A and B)]. Compared with control SOD1-G93A mice, DHT-treated SOD1-G93A mice exhibited 26% more myelinated axons in the phrenic nerves [SOD1-G93A+DHT: 232.3±16.8, *p* = 0.043 (Fig. 6A and B)].

The effect of DHT in protecting nerves innervating hindlimb muscles was also observed by checking the myelinated axon number in the ventral root of the lumbar spinal cord. In SOD1-G93A mice, about a 30% decrease in the total number of myelinated axons was observed at the ventral root of L4 compared with wild-type mice at P120 [SOD1-G93A: 691.2±43.6, WT: 996.5±58.5, *p* = 0.0012 (Fig. 6C and D)]. It has been reported that large caliber axons are preferentially affected by ALS [41], [42], and, indeed, we found a 43% reduction in the number of large caliber axons in SOD1-G93A mice compared with wild-type mice [SOD: 404.2±31.1, WT: 706.5±51.6, *p* = 0.0005 (Fig. 6D)]. Compared with control SOD1-G93A mice, there were 24% more of total myelinated axons in DHT-treated SOD1-G93A mice (859.6±53.4, *p* = 0.013), which contained 36% more of large caliber axons (547.8±22.2, *p* = 0.006, Fig. 6C and D). Conversely, orchidectomized SOD1-G93A mice showed 10% less total myelinated axons (622.3±29.6, *p* = 0.01, Fig. 6C and D) compared with control SOD1-G93A mice.

### DHT improves motoneuron survival in SOD1-G93A mice

**Figure 7:**
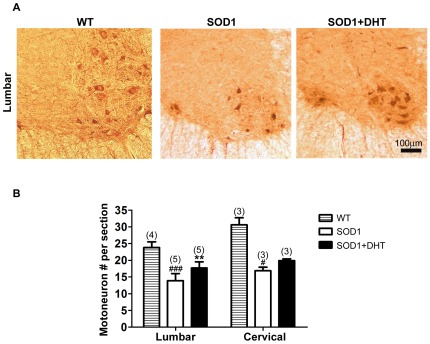
DHT improves motoneuron survival in SOD1 mice. **A:** SOD1 mice were implanted with either a DHT-filled or an empty silastic (control) tube at P75, and the lumbar spinal cord (L) 3-L5 was collected at P120, the symptomatic age. Cross-sectional pictures of the hemi-lumbar spinal cord in WT, control SOD1, and DHT-treated SOD1 mice are shown. **B:** There was a 41% reduction in motoneuron number in the L3–L5 of SOD1 mice (13.9±0.7, n = 5) compared with WT mice at P120 (23.8±0.9, n = 4, *p*<0.0001). DHT-treated SOD1 mice contained 27% more motoneurons (17.7±0.8, n = 5, *p* = 0.0093) compared with control SOD1 mice. In the cervical spinal cord, there were 45% less motoneurons in SOD1 mice (16.8±0.7, n = 3, *p* = 0.015) compared with WT mice at P120 (30.6±1.5, n = 3). There were 18% more motoneurons in DHT-treated SOD1 mice (19.9±0.4, n = 3, *p* = 0.467) compared with control SOD1 mice. Data are mean ± SEM. **p<0.01(compared with control SOD1 mice), #*p*<0.05, ### *p*<0.001 (compared with age-matched WT mice).

To examine whether DHT treatment increased motoneuron survival, we labeled the spinal cord sections with a choline acetyltransferase (ChAT) antibody to visualize motoneurons. We found about a 40% reduction in the number of motoneurons in the lumbar spinal cord (L3–L5) of SOD1-G93A mice compared with wild-type mice at P120 [SOD1-G93A: 13.9±0.7, WT: 23.8±0.9, *p*<0.0001 (Fig. 7A and B)]. Compared with control SOD1-G93A mice, DHT-treated SOD1-G93A mice showed 27% more motoneurons in the lumbar spinal cord [SOD1-G93A+DHT: 17.7±0.8, *p* = 0.0093 (Fig. 7A and B)]. In the cervical spinal cord, which contains motoneurons innervating the diaphragm muscle, we also found 18% more motoneurons in DHT-treated SOD1-G93A mice compared with control SOD1-G93A mice [SOD1-G93A+DHT: 19.9±0.4, SOD1-G93A: 16.8±0.7, *p* = 0.47 (Fig. 7 B)].

### DHT improves motor performance and survival in SOD1-G93A mice

**Figure 8:**
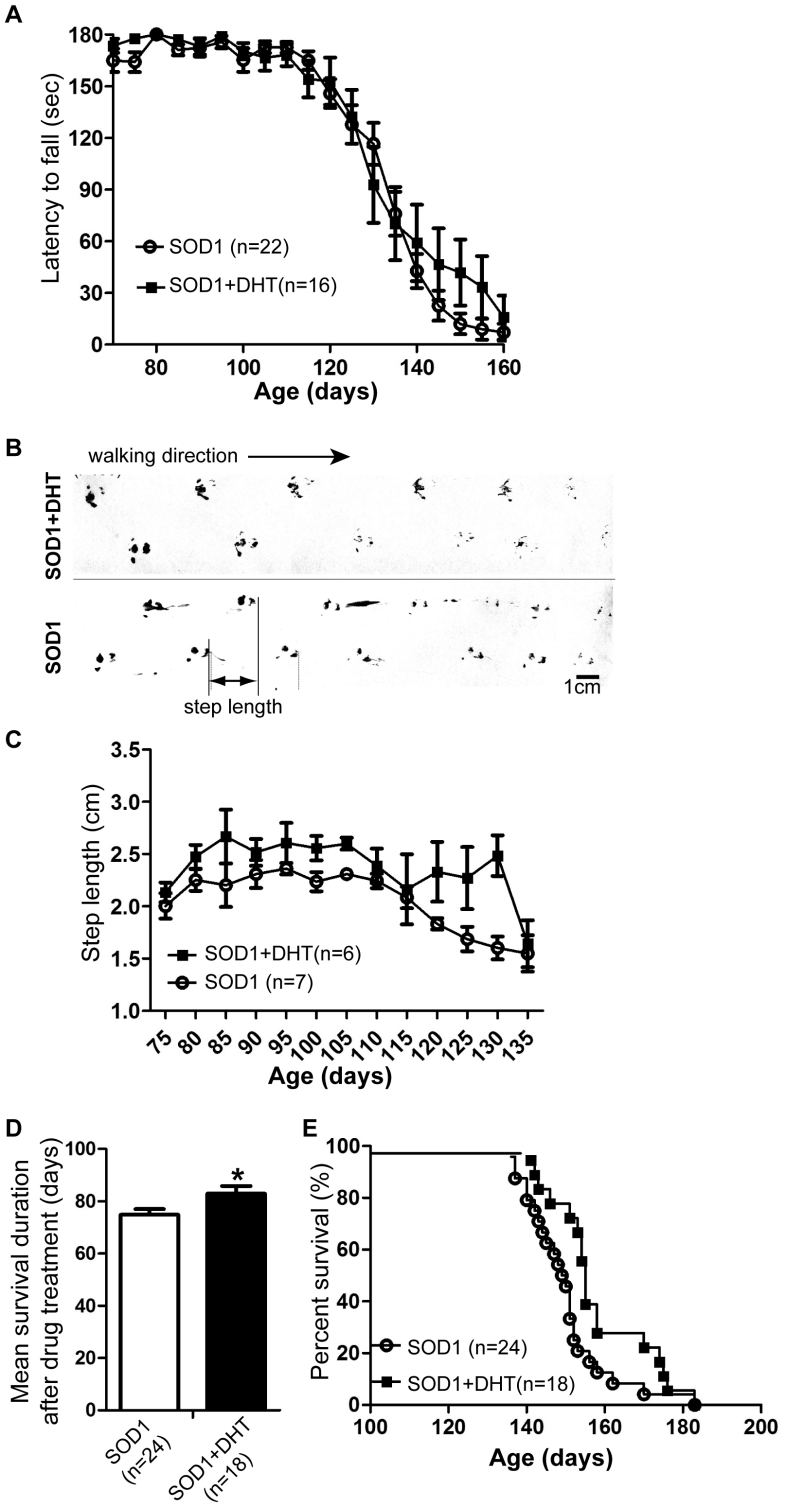
DHT improves motor performances and survival in SOD1 mice. **A:** SOD1 mice were implanted with either a DHT-filled or an empty silastic (control) tube at P75, and their motor performances were assessed every 5 days by the rota-rod test. A mouse was placed on the rotating rod at 11 rpm, and the latency until the mouse fell from the rotating rod was recorded in seconds. DHT-treated SOD1 mice (filled square, n = 16) stayed longer on the rotating rod compared with control SOD1 mice (empty circle, n = 22). Data are mean ± SEM. *p* = 0.043 (2-way ANOVA). **B:** Hindlimbs were placed into non-toxic paints and a mouse was allowed to walk on a 50 cm- length of paper, and its continuous locomotion was recorded. Representative footprints obtained from DHT-implanted or empty tube-implanted SOD1 mice at P100 are shown. DHT-treated SOD1 mouse (top panel) showed a longer step length without a trace of dragging hindlimb compared with control SOD1 mice (bottom panel). **C:** Step length was quantified by dividing walking distance by the number of the steps taken within the measured walking distance. DHT-treated SOD1 mice (filled square, n = 6) showed longer step length compared with control SOD1 mice (empty circle, n = 7). *p* = 0.003 (paired t-test). **D:** DHT-treated SOD1 mice showed significantly increased survival duration after DHT administration by 11% in DHT-treated SOD1-G93A mice (82.9±2.9 days, n = 18) compared with age-matched control SOD1-G93A mice (74.8±2.2 days, n = 24). **E:** DHT-treated SOD1 mice showed significantly increased the overall lifespan by 8 days (filled square, 157.9±2.9 days, n = 18) compared with control SOD1 mice (empty circle, 149.8±2.2 days, n = 24). *p* = 0.0174 (log-rank test).

To determine whether the improved morphological features in DHT-treated SOD1-G93A mice would lead to functional improvement, we tested the effects of DHT treatment in motor behavior and survival of SOD1-G93A mice. In addition to the grip-strength analysis (Fig. 3B), which we previously performed in relation to muscle mass, we further employed rota-rod and footprint analyses as more global assessments of functional outcome. The rota-rod test requires an ability to balance on the rotating rod in addition to muscle strength. Although both DHT-treated and control SOD1-G93A mice showed a similar decline of motor function in the rota-rod test, the gap between two groups increased as disease progressed (Fig. 8A). For example, DHT-treated SOD1-G93A mice stayed 40% longer on the rotating rod compared to control SOD1-G93A mice at P140 (Fig. 8A, *p* = 0.043). To perform footprint analysis, which are used to detect impaired motor behavior [43], the paws of SOD1-G93A mice were placed into non-toxic paints and allowed to walk on a paper. As shown in Fig. 8B, a DHT-treated SOD1-G93A mouse displayed a longer step length compared with a littermate control SOD1-G93A mouse at P100. Analysis of step length illustrates that DHT-implanted SOD1-G93A mice displayed a longer step length compared with control SOD1-G93A mice during disease progression (*p* = 0.003, Fig. 8C). Consistent with the ameliorated motor dysfunction observed in DHT-treated SOD1-G93A mice, the mean survival duration after drug treatment was increased by about 11% in DHT-treated SOD1-G93A mice (82.9±2.9 days) compared with control SOD1-G93A mice (74.8±2.2 days) (Fig. 8D). The overall lifespan of DHT-treated SOD1-G93A mice also showed a slight, but significant increase by 5% (157.9±2.9 days, n = 18), as compared to control SOD1-G93A mice (149.8±2.2 days, n = 24) (*p* = 0.0174; log-rank test, Fig. 8E). In summary, DHT treatment delayed motor impairment as assessed by the rota-rod, grip-strength, and foot-print analyses, and moreover extended lifespan in SOD1-G93A mice.

## Discussion

The present study demonstrated that DHT alleviated the pathological symptoms found in the skeletal muscle of SOD1-G93A mice, which includes muscle atrophy and weakness. In addition, DHT ameliorated neuromuscular pathology and degeneration in axons and motoneurons. Moreover, these improved morphological characteristics were linked with improved motor behaviors and extended lifespan. The beneficial effect of DHT might be accomplished through several possible mechanisms including its effect on muscle, muscle-mediated retrograde transport, and its direct effect on motoneurons.

### The anabolic effect of DHT on skeletal muscle

Although muscle atrophy and weakness are the clinical symptoms linked with functional capacity of ALS patients, there is currently no effective treatment for these clinical symptoms. Since increased motoneuron survival is not sufficient to maintain NMJ innervation and motor function [8], [44], a therapy that can intervene in muscle symptoms needs to be considered for a practical benefit to patients. In the present study, we demonstrated that increased androgen level by DHT treatment can ameliorate muscle atrophy and also improve muscle strength in SOD1-G93A mice. The protective effect of androgen in maintaining muscle mass and function was also observed in mice that underwent orchidectomy, which results in muscle wasting and weakness [13].

Contrary to the beneficial effects of androgens, decreasing androgen was suggested as a therapy for another late-onset motoneuron disease called spinal bulbar muscular atrophy (SBMA), which also demonstrates muscle weakness and atrophy [45], [46]. Although SBMA and ALS share some clinical symptoms, unlike ALS, SBMA expresses aberrant androgen receptor (AR), which contains an expansion of the polyglutamine track caused by CAG repeats in the first exon of the AR gene [47]. Since androgen induces toxic nuclear accumulation of mutant ARs [48], androgen treatment aggravates symptoms of SBMA and would cancel out potential beneficial effects of androgen that we observed in the present study.

Besides AR, androgens also modulate muscle through an AR-independent pathway. Androgen withdrawal through castration resulted in a 200% decrease in GN muscle weight in mice lacking AR in limb muscles [49]. It was suggested that reduced expression of IGF-1 upon castration is responsible for the decrease in muscle mass. In fact, IGF-1 has been suggested as a candidate molecule that mediates androgen-induced muscle growth [50], [51]. Muscle-specific overexpression of IGF-1 and intramuscular injection of IGF-1 expressing viral vector demonstrated marked effect in increasing skeletal muscle [36], [52]. Since we observed that DHT treatment in SOD1-G93A mice increased IGF-1 expression in skeletal muscle (Fig 4A), it is possible that DHT ameliorated muscle atrophy through IGF-1 and its downstream signaling pathways including phosphatidylinositol 3-kinase/Akt/mTOR kinase cascade, which lead to muscle growth [34].

### DHT-mediated increase of IGF-1 in muscle as a neurotrophic source through retrograde transport

In addition to the myotrophic effect of IGF-1, muscle-derived IGF-1 can be retrogradely transported, and may contribute to motoneuron survival. Indeed, IGF-1 is one of the most potent neurotrophic factors tested in ALS model mice. Intramuscular injection of IGF-1 carried by adeno-associated virus enabled successful retrograde transport of IGF-1 to motoneurons, and demonstrated pronounced effects in protecting motoneurons as well as in delaying disease progression of ALS [52], [53]. Moreover, restricted expression of IGF-1 in skeletal muscle also protected motoneurons through retrograde transport and increased lifespan in ALS model mice [54], which further confirms the contribution of muscle in ALS disease progression. Compared to the preclinical studies utilizing viral vectors encoding IGF-1 for an efficient and prolonged supply, this approach may not be easily translated to patients due to difficulties regarding gene therapy. Although direct injection of IGF-1 is a possible option, no significant effect in survival had been found through this approach mainly due to the short half-life of protein [55], [56]. Moderate success was made through intrathecal administrations of IGF-1 to patients, but repetitive invasive injections in the spinal cord are required for chronic delivery of IGF-1 [57]. Considering the difficulties in delivery of IGF-1, endogenous increase of IGF-1 through DHT treatment may have a significant meaning for translational studies. Based on our study showing that DHT induced expression of IGF-1 in skeletal muscle in SOD1-G93A mice, DHT treatment might be an indirect but relatively non-invasive method to increase endogenous IGF-1 expression in muscle, which can be retrogradely transported to motoneurons to promote neuronal survival. It is possible that there might be an additional benefit of retrogradely transported trophic factor as compared to the centrally delivered factor because overexpressed GDNF in muscle increased the motoneuron survival and delayed disease progression while overexpressed GDNF in astrocytes failed to show beneficial effects in ALS model mice [58]. Similarly, viral vector-mediated administration of IGF-1 directly into the spinal cord failed to elicit a survival effect in a rat model of ALS despite a protective effect on motoneuron survival [59]. Therefore, there might be an additional benefit of retrograde transport, which might be that muscle-derived trophic factors are likely to benefit both motoneurons and muscle.

### The neuroprotective effect of DHT

Besides the potential neuroprotective effect mediated by IGF-1, androgens demonstrate effects in improving neuronal survival through androgen receptors (ARs) [60]. Motoneurons in the spinal cord express high level of ARs [15], and androgens increase motoneuron survival in several experimental conditions. Androgens increase motoneuron survival rate from 50% to 90% after nerve injury in the avian embryonic lumbar spinal cord [61]. Similarly, increased motoneuron survival through androgen treatment was observed in organotopic culture of the spinal cord [62], as well as in neuroblastoma and motoneuron hybrid cells [63]. Even in SBMA model mice expressing toxic mutant ARs, normal ARs still play an important role in neuroprotection, thus removing normal ARs can accelerate the neurodegeneration in SBMA [64].

It is worth noting that the ventral horn of the spinal cord expresses high levels of 5-α reductase [15], [65], which converts testosterone into DHT. Therefore, it is possible that DHT might be the androgen inducing motoneuron survival in the spinal cord, and we observed that DHT-treated SOD1-G93A mice contained more motoneurons in the spinal cord than control SOD1-G93A mice (Fig. 7). It is likely that DHT and AR-mediated pathway together with muscle-derived IGF-1-mediated pathway contributed to increased motoneuron survival in SOD1-G93A mice. In regard of a recent study demonstrating that DHT can be converted to 3beta-Adiol, which activates the estrogen receptor beta [66], we cannot exclude a possibility that ER-mediated pathways might also contribute to the neuroprotective effect of DHT.

Furthermore, androgen is known to increase the regeneration rate of axons after peripheral nerve injury caused by axotomy [16]. Therefore, improved NMJ innervation in the hindlimb of DHT-treated SOD1-G93A mice might be mediated through the beneficial effect of DHT on axonal regeneration. The increased myelinated axon number observed in the nerves of DHT-treated SOD1-G93A mice (Fig. 6) is also possibly mediated by DHT-induced axonal regeneration, which might counteract dying-back axonal degeneration.

### Concerns regarding androgen treatment

Despite improved morphological phenotypes in skeletal muscle, axons, and motoneurons observed in DHT-treated SOD1-G93A mice, we found a relatively small effect on survival. It is possible that DHT treatment may result in decreased fat storage, which can be used as an energy source. In fact, hypermetabolism and associated defects in energy homeostasis have been implicated in ALS [67], and therefore high fat diet exerts beneficial effects in delaying the disease progression of ALS [68], [69]. However, androgens are known to reduce fat through inhibiting adipogenesis and increase muscle by enhancing myogenesis [70], [71]. Since DHT might affect fat storage, intake of a high fat diet might be desirable to compensate the possible loss of fat storage as an energy source.

Although molecular mechanisms need to be further elucidated, considering the beneficial effects of DHT on multiple cell types including muscle and neurons, DHT might be a potential candidate as a multi-systemic drug for ALS involving neuronal and non-neuronal cells in disease progression. In support of a notion that targeting multiple cell types implicated in ALS might be a potential therapeutic strategy, we have also demonstrated that trichostatin A, which exerts beneficial effects on neurons, glia, and muscle, delays the disease progression of ALS [72]. Considering the fact that there is no effective drug for ALS patients to improve pathological symptoms, the beneficial effect of DHT illustrated in the present study may provide an initial step toward a possible therapy for ALS. However, several concerns remain regarding adverse side effects of chronic DHT treatment. Since DHT may increase the incidence of benign prostate hyperplasia due to the proliferative activity in the prostate [73], alternative androgens that have almost no effect on the prostate such as selective androgen receptor modulators (SARMs) [74] can be tested to see whether they can replicate the effects of DHT in ALS. Since ALS is a progressive disease that usually causes death within 2–3 years, if the side effects are minor with DHT or SARMs, these treatments might be tolerable.
